# Preoperative serum CD26 levels: diagnostic efficiency and predictive value for colorectal cancer

**DOI:** 10.1054/bjoc.2000.1410

**Published:** 2000-11

**Authors:** O J Cordero, D Ayude, M Nogueira, F J Rodriguez-Berrocal, M Paez de la Cadena

**Affiliations:** Department of Biochemistry and Molecular Biology, Faculty of Biology, University of Santiago de Compostela, Santiago de Compostela, 15706; Department of Biochemistry, Genetics and Immunology, Faculty of Sciences, University of Vigo, Vigo, 36280, Spain

**Keywords:** colorectal cancer, serum CD26, CD26/DPP-IV, diagnosis, tumour recurrence

## Abstract

CD26 is an ectoenzyme with dipeptidyl peptidase IV activity expressed on a variety of cell types. Although the function of the high concentration of serum-soluble CD26 (sCD26) is unknown, it may be related to the cleavage of biologically active polypeptides. As CD26 or enzymatic activity levels were previously associated with cancer, we examined the potential diagnostic and prognostic value of preoperative sCD26 measurements by ELISA in colorectal carcinoma patients. We found a highly significant difference between sCD26 levels in healthy donors (mean 559.7 ± 125.5 μg l^–1^) and cancer patients (mean 261.7 ± 138.1 μg l^–1^) (*P* < 0.001). A cut-off at 410 μg l^–1^ gave 90% sensitivity with 90% specificity which means that the diagnostic efficiency of sCD26 is higher than that shown by other markers, particularly in patients at early stages. Moreover, sCD26 as a variable is not related with Dukes’ stage classification, age, gender, tumour location or degree of differentiation. With a follow-up of 2 years until recurrence, preliminary data show that sCD26 can be managed as a prognostic variable of early carcinoma patients. In addition, the origin of sCD26 is discussed. © 2000 Cancer Research Campaign


British Journal of Cancer (2000) 83(9), 1139–1146
© 2000 Cancer Research Campaign
doi: 10.1054/ bjoc.2000.1410, available online at http://www.idealibrary.com on IDEAL®

### Preoperative serum CD26 levels: diagnostic efficiency and predictive value for colorectal cancer

**OJ Cordero[1], D Ayude[2], M Nogueira[1], FJ Rodríguez-Berrocal[2] and M Páez de la Cadena[2]**

[1]Department of Biochemistry and Molecular Biology, University of Santiago de Compostela, Faculty of Biology, 15706 Santiago de Compostela; [2]Department of Biochemistry, Genetics and Immunology, University of Vigo, Faculty of Sciences, 36280 Vigo, Spain

**Summary** CD26 is an ectoenzyme with dipeptidyl peptidase IV activity expressed on a variety of cell types. Although the function of the high concentration of serum-soluble CD26 (sCD26) is unknown, it may be related to the cleavage of biologically active polypeptides. As CD26 or enzymatic activity levels were previously associated with cancer, we examined the potential diagnostic and prognostic value of preoperative sCD26 measurements by ELISA in colorectal carcinoma patients. We found a highly significant difference between sCD26 levels in healthy donors (mean 559.7 ± 125.5 μg $l^{-1}$) and cancer patients (mean 261.7 ± 138.1 μg $l^{-1}$) ($P < 0.001$). A cut-off at 410 μg $l^{-1}$ gave 90% sensitivity with 90% specificity which means that the diagnostic efficiency of sCD26 is higher than that shown by other markers, particularly in patients at early stages. Moreover, sCD26 as a variable is not related with Dukes' stage classification, age, gender, tumour location or degree of differentiation. With a follow-up of 2 years until recurrence, preliminary data show that sCD26 can be managed as a prognostic variable of early carcinoma patients. In addition, the origin of sCD26 is discussed. © 2000 Cancer Research Campaign

**Keywords**: colorectal cancer; serum CD26; CD26/DPP-IV; diagnosis; tumour recurrence

Malignant transformation from normal to cancerous tissue is associated with cell-surface glycoprotein and glycolipid modifications (Hakomori, 1989). These glycoconjugates can be released in the circulation through increased cell turnover, secretion or shedding from the malignant cells and have been considered as potential tumour markers for helping in screening, diagnosis, staging, prognosis and monitoring of cancer therapy (Cohn et al, 1986). The protease dipeptidyl peptidase IV (DPP-IV, EC 3.4.14.5.) is a 110 000 MW cell-surface glycoprotein expressed on a variety of cell types, particularly melanocytes, epithelial cells (Iwata and Morimoto, 1999) and lymphocytes, where DPP-IV is necessary for normal immune function (see review, De Meester et al, 1999) and was assigned to the CD26 cluster. CD26 is a functional receptor for collagen (Bauvois, 1988; Dang et al, 1990) and was also recently identified as the adenosine deaminase binding or complexing protein (ADAbp, ADCP) (Kameoka et al, 1993; De Meester et al, 1994). Significant levels of DPP-IV activity have been shown to exist in plasma, serum and urine (Sharpé et al, 1988). The MW of serum CD26 (sCD26) suggests that it is originated by a shedding of membrane CD26 (Iwaki-Egawa et al, 1998). sCD26 can cleave $NH_2$-terminal dipeptides from polypeptides with either L-proline or L-alanine at the penultimate position (Fleischer, 1994). Many biologically active polypeptides have this sequence, for example substance P, chorionic gonadotropin, monomeric fibrin, promellitin (Bauvois et al, 1992) and regulatory peptides such as glucagon-like peptides 1 and 2 (Drucker et al, 1997). A proline residue is also present at the P1 position in many cytokines, such as IL-1β, IL-2, IL-6, and G-CSF (Ansorge et al, 1991). CD26 enzymatic activity also affects TNF-α (Bauvois et al, 1992), neuropeptides such as Y and YY (Medeiros and Turner, 1994) and chemokines activity (Oravecz et al, 1997; Proost et al, 1998).

CD26, when known as ADCP, was described to be consistently associated with cancer. The loss of DPP-IV expression during malignant transformation has been best characterized in melanocytic cells, although a role for DPP-IV in regulating the malignant phenotype had not been shown until very recently (Iwata and Morimoto, 1999; Wesley et al, 1999). A deficiency in solubilized CD26 was reported in total homogenates of tumours of colon, kidney, lung and liver (Ten Kate et al, 1985; 1986*a*), as well as in different transformed or cancer-derived cell lines (Ten Kate et al, 1986*b*). On the contrary, cell-surface CD26 expression has been correlated with disease aggressiveness of T and B cell lymphomas and leukaemias, follicular cell-derived thyroid carcinomas and basal cell carcinomas (reviewed in Iwata and Morimoto, 1999). In addition, serum DPP-IV activity was increased in patients with hepatic cancer (Hino et al, 1975; Kojima et al, 1987), and decreased in patients with blood, solid and oral (Fujita et al, 1977; Mogi et al, 1986; Uematsu et al, 1996) cancer. From these data, it seems helpful to fully evaluate the potential significance of serum CD26 as a colon carcinoma (which remains a major medical problem) (American Society of Clinical Oncology, 1996; 1998) diagnostic and prognostic marker.

### MATERIALS AND METHODS

### Patients

Preoperative blood and primary tumour samples were collected between January 1994 and December 1997 from 110 potentially curable patients (56 females, 54 males, mean age 68, range 34–88), operated for colorectal cancer (74 colon, 36 rectum) in the

*Received 23 February 2000*
*Revised 1 June 2000*
*Accepted 28 June 2000*

*Correspondence to:* OJ Cordero

Complexo Hospitalario Xeral-Cíes, Vigo, Spain. Twenty-three patients from the same hospital with other diseases were also studied: nine patients with gastrict tract carcinomas, five patients with Crohn's disease, five patients with benign pathology of the gastric tract and four patients with blood cell cancer. The control group, consisting of 52 healthy blood donors (23 females, 29 males) was provided by the Centro de Transfusion de Galicia throughout 1997. Some data of the population samples came from other studies (Fernández-Rodríguez et al, 2000; Ayude et al, 2000).

For prognostic studies, no patient receiving adjuvant therapy either preoperatively or postoperatively was included. Patients with family adenomatous polyposis coli, inflammatory bowel disease, or previous colorectal cancer were not included for review. The presence of metastasis or the failure to resect all the tumour deemed the resection palliative and these patients were excluded from analysis, as were those who died within 30 days of surgery. Thus, only 87 potentially cured patients with Dukes' stages A–C were followed-up for 2 years until recurrence.

### Preparation of samples

The drawn blood was allowed to coagulate at room temperature and centrifuged at 2000 ***g*** for 15 min. The sera were stored at –85°C until used. All adenocarcinoma samples were processed for regular pathological and histological examination.

### Immunoassays

The concentration of serum CD26 and carcinoembryonic antigen (CEA) were analysed using specific immunoassays (human soluble CD26 ELISA Kit from Bender Medsystems, Vienna, Austria, and Enzymun-Test CEA from Boehringer Mannheim, Germany). ELISAs were performed according to the manufacturer's instructions: mean values of duplicated measurements were calculated and a sigmoid-shaped standard curve was determined by simultaneously analysing a dilution series of standard samples. Specificity of sCD26 system was evaluated by the manufacturer for several circulating factors of the immune system and no cross-reactivity was detected. No cross-reactivity of anti-sCD26 Abs with rheumatoid factor was found in a previous study (manuscript submitted).

### Prognostic predictors

Five clinical and pathological variables were evaluated according to the following definitions: age, sex, tumour location, stage, and degree of differentiation. Age was recorded in years at time of operative intervention; for statistical analysis, patients were grouped into two categories, ≤ 50 and > 50 years old. For the recurrence study, the cut-off chosen was ≤ 76 or > 76, to better discriminate the two groups. Recurrence pattern based on patients' sex was also analysed. The site of the primary lesion was determined from the operative report. The large-bowel was divided into two regions for statistical analysis: colon (including right and left colon, and sigma) and rectum (lesions were considered rectal if their origin was below the peritoneal reflection). The stage of disease was originally reported using Dukes' classification (invasive and metastatic potential of tumours) (Dukes, 1932) as determined in the original operation. The degree of differentiation as described by the pathologist in the original operation was recorded and classified into three categories: well, moderately and poorly differentiated lesions.

### Statistical methods

Normal distribution was assessed using the Kolmogorov-Smirnov test. Variance homogeneity was evaluated by the Levene test. The statistical significance of the results was assessed using a nonpaired Student's *t*-test or Mann–Whitney U test performed in the SPSS program for Windows (Release 7.5.2S, or 8.0). Statistical comparisons among groups were made by Kruskal–Wallis and ANOVA tests, according to the Levene test results, respectively. A cut-off value for CD26 was determined using receiver operating characteristics (ROC). ROC curves are plots of the percentage true-positives (sensitivity) against the percentage false-positives (100-specificity) for multiple thresholds (Beck and Shultz, 1986; Zweig and Campbell, 1993). In order to evaluate the impact of each variable over the disease-free interval, a postoperative follow-up of the patients was performed. Kaplan–Meier curves (Kaplan and Meier, 1958) were constructed, with colorectal cancer-related recurrence as the primary end-point. Differences in disease-free survival (DFS) among groups were assessed by log-rank analysis. $P$ values ≤ 0.05 were considered statistically significant.

### RESULTS

### sCD26 levels in serum of healthy donors and patients with colorectal cancer

Soluble CD26 concentration (μg $l^{-1}$) was determined in 110 sera from patients with colorectal cancer and 52 control sera from healthy donors. Data from both populations follow a normal distribution. The concentration of sCD26 was dramatically impaired in many colorectal cancer patients (261.65 ± 138.07 μg $l^{-1}$, range 56–980 μg $l^{-1}$) with respect to control donors (559.65 ± 125.52 μg $l^{-1}$, range 273–863 μg $l^{-1}$), on average by 53% ($P < 0.001$).

Statistical analysis after this comparison gave the results shown in Table 1. There were no significant differences between the two sex- and age-groups for both donor and patient samples, which is particularly interesting in the second case, due to the strong difference between donor and patient samples in the number of recruited individuals for both age-groups. These data agree with the fact that DPP-IV activities did not differ significantly with age (Hino et al, 1975).

### Relationship between preoperative serum sCD26 levels and clinicomorphologic features of tumours

Table 2 compares by the Kruskal–Wallis test preoperative serum sCD26 levels and the Dukes' stages from patients with colorectal adenocarcinoma. In addition, statistical comparisons between groups were made by using the Mann–Whitney U test, as there was not variance homogeneity. By these analyses, there is no difference in the preoperative serum activity of sCD26 among Dukes' stages. The possible association between preoperative serum sCD26 levels and age, sex, tumour location and degree of histologic differentiation of tumours was also examined. According to our data (Tables 1, 2 and 3) none of these properties was correlated with preoperative serum sCD26 levels.

 *© 2000 Cancer Research Campaign*

**Table 1** Serum CD26 concentration in donors and patients with colorectal cancer

| Case | *n* | Mean ± SD ($\mu g\ l^{-1}$) | SEM | Range | Student's *t*-test |
|---|---|---|---|---|---|
| **Donors** | **52** | **559.65 ± 125.53** | **17.41** | **273–863** | |
| Sex | | | | | |
| Men | 23 | 584.70 ± 113.93 | 23.76 | | |
| | | | | | NS |
| Women | 29 | 539.79 ± 132.58 | 24.62 | | |
| Age | | | | | |
| < 50 years | 39 | 557.36 ± 126.31 | 20.23 | | |
| | | | | | NS |
| ≥ 50 years | 13 | 566.54 ± 127.95 | 35.49 | | |
| | | | | | ***P* < 0.001** |
| **Tumoural** | **110** | **261.65 ± 138.07** | **12.45** | **56–980** | |
| Sex | | | | | |
| Men | 56 | 235.77 ± 101.71 | 13.59 | | |
| | | | | | NS |
| Women | 54 | 273.52 ± 155.92 | 21.22 | | |
| Age | | | | | |
| < 50 years | 9 | 218.56 ± 77.42 | 25.81 | | |
| | | | | | NS |
| ≥ 50 years | 101 | 257.49 ± 135.50 | 13.48 | | |

NS = not significant; *P* = statistical significance. The age cut-off point (50 years) was chosen because it can be used in both donor and tumoural groups, facilitating comparison

**Table 2** Relationship between the levels of sCD26 and the Dukes' stage classification

| Dukes' stage | *n* | Mean ± SD ($\mu g\ l^{-1}$) | SEM | Median | Range | Kruskal–Wallis test |
|---|---|---|---|---|---|---|
| A | 12 | 295.33 ± 152.34 | 43.98 | 292 | 78–663 | |
| B | 55 | 226.67 ± 93.06 | 12.55 | 205 | 100–438 | NS |
| C | 29 | 251.48 ± 95.45 | 17.73 | 248 | 78–522 | |
| D | 14 | 333.50 ± 243.10 | 64.97 | 312 | 56–980 | |

NS = not significant

### sCD26 levels in serum of patients with other related carcinomas and benign diseases

The level of sCD26 was also determined in serum of 23 patients with related diseases other than colorectal cancer, preliminarily to check the specificity of our finding. These patients were included in three different groups: patients with gastric-tract carcinomas (GC, $n = 9$), patients with blood cell cancer (BCC, $n = 4$) and patients with Crohn's disease or with benign pathology of the gastric tract (BPI, $n = 10$). Results shown in Figure 1 suggest that sCD26 levels discriminate well colorectal carcinoma from gastric (585 ± 148 $\mu g\ L^{-1}$) and blood cell cancer (663 ± 196 $\mu g\ l^{-1}$), and that a study on sCD26 in different BCCs could clear the origin of sCD26 because its levels are enhanced in some but not in other BCCs. Curiously, the majority of BPI show low sCD26 levels whereas Crohn's patients presented a very irregular distribution.

### Diagnostic efficiency of sCD26 preoperative serum levels

Receiver operating characteristics (ROC) curves for sCD26 and CEA are represented in Figure 2. The corresponding sensitivities at different specificity levels for sCD26 are provided in Table 4. The ideal cut-off point for diagnostic value of sCD26 was determined by random selection of outstanding points from the ROC curve. The best efficiency (90%) was obtained with the point 410 $\mu g\ l^{-1}$, representing a sensitivity of 90% with a specificity of 90%. Even with a specificity of the 100% the efficiency is good (71%). This curve revealed that the diagnostic efficiency of sCD26 levels is higher than that shown by CEA, the more extensively used in health services, which showed an efficiency of the 69%. We also studied the diagnostic efficacy of sCD26 in Dukes' stages A, B, C and D patients. Figure 3 shows that the sensitivity is enhanced in the A, B and C stages, whereas was impaired in the Dukes' stage D, in which CEA levels diagnosed better.

### sCD26 as prognostic predictor in colorectal cancer

From the 110 patients with colorectal adenocarcinoma included in this work, only 87 potentially cured patients with Dukes' stages A–C were followed-up for at least 1 year or until recurrence. After a mean postoperative follow-up period of 22 months, recurrence appeared in 18 patients. Disease-free survival time was 39 months (mean confidence interval, 35–43, maximum 49, $n = 87$) and the percentage of tumoural recurrence was 21.1%. The survival curve of the total potentially cured patients included in this study is plotted in Figure 4A. The same study was performed considering only patients in Dukes' stage B (Figure 4B) or C (data not shown) (there was not enough data for A), finding a non-significant result and a particularly bad behaviour of sCD26 as a prognostic marker in Dukes' stage C. Table 5 shows the results of a univariate survival analysis of patients stratified into groups by biochemical, clinical and pathological features. From these parameters, only the Dukes' stages, as currently described, and sCD26 were related to

© 2000 Cancer Research Campaign 

**Table 3** Relationships between preoperative sCD26 levels and the degree of tumour differentiation or location

| | *n* | Mean ± SD (µg l⁻¹) | SEM | ANOVA |
|---|---|---|---|---|
| Degree of differentiation | | | | |
| Good | 10 | 257.30 ± 86.28 | 27.28 | |
| Moderate | 90 | 247.83 ± 112.92 | 11.90 | NS |
| Poor | 9 | 235.00 ± 141.82 | 47.27 | |
| Location | | | | **Student's *t*** |
| Colon | 74 | 251.24 ± 141.69 | 16.47 | |
| | | | | NS |
| Rectum | 36 | 260.58 ± 110.69 | 18.45 | |

NS = statistically not significant. With regard to the degree of differentiation, information from one patient was lost

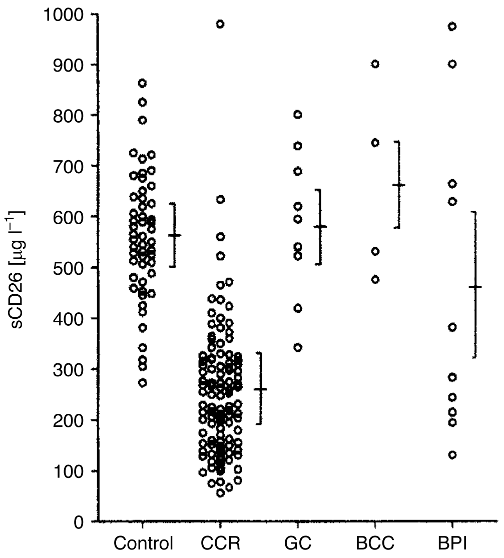

**Figure 1** Serum CD26 (µg l⁻¹) in healthy donors and patients with: colorectal cancer (CRC), gastric-tract carcinoma (GC), Crohn's disease and other benign pathology of intestinal tract (BPI), and blood cell cancer (BCC). The vertical lines represent the SD values

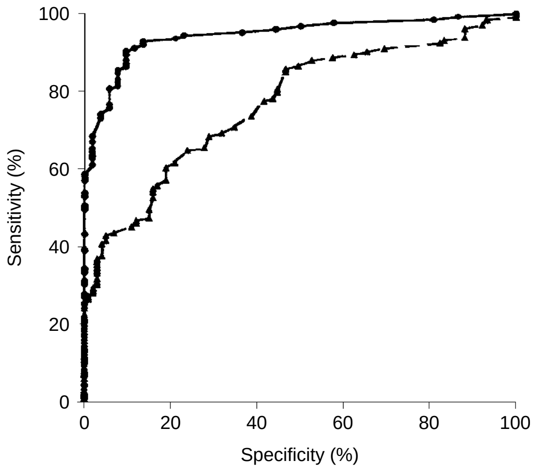

**Figure 2** ROC curves for the serum levels of CD26 and CEA

long-term outcome in this study. For sCD26, two groups of patients could be differentiated choosing the cut-off in the 60th percentile (250 µg l⁻¹), the log-rank test giving a $P = 0.0346$. Data from the survival curves of Figure 4 and the fact that age and degree of differentiation (Table 5) are near to significance in this work, are encouraging us to study a larger follow-up period.

### DISCUSSION

Cell-surface proteases participate in malignant transformation and cancer progression by facilitating invasion and metastasis. However, they may also have the opposite effect. This is the case for DPP-IV/CD26 (Werb, 1997; Iwata and Morimoto, 1999). The diverse biological functions of CD26 (De Meester et al, 1999; Iwata and Morimoto, 1999) may be responsible in part for the different roles of CD26 in various clinical settings. In melanoma cells, where membrane expression of DPP-IV is, as well as in CRC, lost or altered (Ten Kate et al, 1986*a*; Morrison et al, 1993), it has been demonstrated that the inducible translation of CD26 reverses the malignant phenotype. It is suggested that DPP-IV enzymatic activity degrades growth factors (unknown autocrine factors, although candidates should be among those cited in the introduction) required for survival of tumour cells.

Surprisingly, little information is available about the physiological activity of sCD26 in spite of its presence at relatively high concentrations in serum (~ 600 µg l⁻¹) of healthy donors. Studies like this should unravel the kind of cells which shed membrane CD26 to the serum. For example, as membrane CD26 is lost in hepatocellular carcinoma (Ten Kate et al, 1985; Iwata and Morimoto, 1999; Perner et al, 1999) and DPP-IV is increased in patients with hepatic cancer (Kojima et al, 1987) as well as in many studies of hepatic regeneration, liver epithelia is one of the suggested sCD26 sources. In this case, sCD26 levels would correlate with cell proliferation. This is clearly not the case for colorectal carcinoma as well as, our data suggest, for other pathologies such as the GC group. The studies on the membrane CD26 in human CRC (Ten Kate et al, 1985; 1986*b*) found a loss of expression in only 11% of the patients, and decreases in a third of the patients. Although this and our study, in which 58% of patients had lower values than the minimum range of normal donors, cannot be directly correlated, it is easily deduced that loss of membrane CD26 and enhancement of sCD26 are not correlated in

© 2000 Cancer Research Campaign

**Table 4** Comparison of three cut-off points for sCD26 (μg $l^{-1}$) in the detection of colorectal cancer

| Cut-off points | Sensitivity (%) | Specificity (%) | Positive predictive value (%) | Negative predictive value (%) | Efficiency (%) |
|---|---|---|---|---|---|
| 270 μg $l^{-1}$ | 59 | 100 | 100 | 50 | 71 |
| 330 μg $l^{-1}$ | 80 | 94 | 97 | 67 | 85 |
| 410 μg $l^{-1}$ | 90 | 90 | 96 | 80 | 90 |

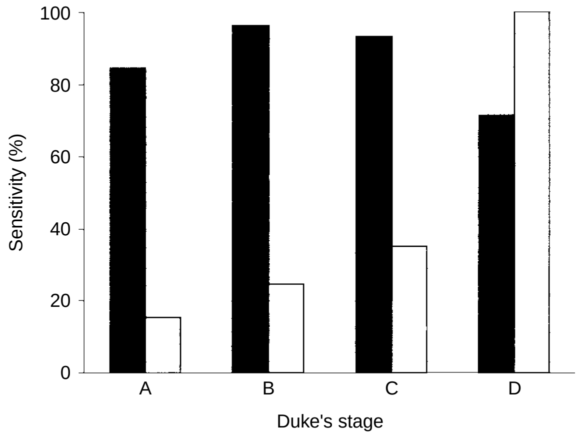

**Figure 3** Sensitivity of sCD26 and CEA serum levels for the diagnosis of colorectal cancer by Dukes' stages

CRC. In addition, we found a lack of any direct correlation of sCD26 with tumour location, degree of histologic differentiation, kind of metastasis or Dukes' stages. In conclusion from all these data, 53% impairment in sCD26 in colorectal cancer does not seem to be originated by alteration of CD26 on CRC tumour cells.

At least two possibilities arise from this conclusion: that CRC, as well as BPI but not GC, is associated with an impairment of the usual hepatic function, or the drop in sCD26 levels is related to the immune system status. For the first case, no data have been published up to now. In fact, as CRC metastases were each all located in liver, an improvement in sCD26 levels should be expected, as well as a correlation with the kind of metastasis. However, a cross-talk between the lymphoid lineage and malignant tumours in vivo have been long discussed (Shibuya-Saruta et al, 1996; Gruss et al, 1997; Nano et al, 1997; Iwata and Morimoto, 1999) and some data about the immune defective antitumour response in CRC have been described before, including a defect in IL-12 production (O'Hara et al, 1998), which is a well-known CD26 up-regulator (Cordero et al, 1997) on T cells. In oral cancer patients, in which around a 50% decrease in serum DPP-IV activity has been reported, a correlation between sCD26 and CD26+ T was found, and the number of T lymphocytes and PBL and the amount of CD26 in T lymphocyte plasma membranes were significantly less than in healthy subjects (Uematsu et al, 1996; 1998). Both possibilities can explain the donor-dependent variations, but the second one seems more probable in the cases of hemicolectomy or rectoragy (some of the lower values of the BPI group), except the Dukes' stage D cases with high sCD26 values, perhaps associated with a high proliferative (hepatic) cellular state, or with a later activation of PBL as suggested in CRC and ovarian tumours for the soluble CD44v6 glycoprotein (Sliutz et al, 1995; Yamane et al, 1999). It seems, then, necessary to collect the lymphocyte count and other immune parameters of the patients in future studies of CRC. As some studies are showing that sCD26 therapy enhances the immune function in some pathological conditions such as AIDS (Schmitz et al, 1996), it might be interesting to analyse if CRC patients may well benefit from exogenous sCD26 treatment.

Very recently, the clinical utility of serum DPP-IV/CD26 activity measurements was tested in adult and paediatric patients with hepatobiliary diseases and in liver transplant recipients. The results established elevated serum DPP-IV activity as a clinically useful marker of cholestasis and demonstrate that DPP-IV levels do not change in metastatic bone disease (Lakatos et al, 1999; Perner et al, 1999) nor in allergic asthmatics, inhaling glucocorticoids or not (Van Der Velden et al, 1999). Higher activities were also found in serum of patients with osteoporosis, probably related to its severity (Gotoh et al, 1988). However, reduced peptidase can be found in healthy smokers (Van Der Velden et al, 1999), alcohol-dependent (Maes et al, 1999) and major depressive (Maes et al, 1997) donors. These last drops are also in agreement with an impaired immune response. The studies described before show alterations in the levels of the serum DPP-IV enzymatic activity. To our knowledge, only one study reported a decrease of protein sCD26 (but to a lesser extent than ours) in oral cancer patients (Mogi et al, 1986), although the authors measured a higher quantity of protein than we by using the commercial kit. In addition, some of our data are not in accordance with published measurements of DPP-IV enzymatic activities in some diseases such as GC (lower levels than in normal subjects) (Hino et al, 1975), or in some patients of the BCC group (Fujita et al, 1977). As at least one different serum protein accounting for the DPP-IV activity (Duke-Cohan et al, 1996) as well as new discovered cellular proteins with DPP-IV activity (Pangalos et al, 1999; Underwood et al, 1999) have been described, these facts should encourage more complete studies at the protein level on different pathologies. Therefore, although we found a normal distribution for sCD26 levels in both samples of Spanish populations, which points to a unique locus for the CD26 gene, a possible effect of race on the absolute levels detected in this study cannot be excluded.

Establishing the diagnosis at an early stage in colorectal cancer, with a simple biochemical index, is a current subject of research in clinical oncology. The CEA levels are the marker of reference in this neoplasia, although not recommended as diagnostic test for CRC (American Society of Clinical Oncology, 1996). Our curve revealed that sensitivities of sCD26 were higher at different specificity levels than those of CEA, as well as efficiency. Moreover, when the diagnostic sensitivity of sCD26 and CEA by Dukes'

*© 2000 Cancer Research Campaign*

**Table 5** Univariate analysis of the disease-free survival (DFS) according to the classical clinicopathological and biochemical features of this study

| **Factor** | **Category** | ***n*** | **DFS (months)** | **Tumoural recurrence (%)** | **Log-Rank test (*P*)** |
|---|---|---|---|---|---|
| Age | | | | | |
| | ≤ 76 years | 67 | 41.30 | 16.42 | 0.0561 |
| | > 76 years | 20 | 29.55 | 35.00 | |
| Sex | | | | | |
| | Male | 47 | 33.49 | 19.15 | 0.8103 |
| | Female | 40 | 39.82 | 22.50 | |
| Location | | | | | |
| | Colon | 59 | 39.93 | 16.95 | 0.1383 |
| | Rectum | 28 | 32.35 | 28.57 | |
| Degree of differentiation | | | | | |
| | Good | 5 | 29.98 | 20.00 | |
| | Moderate | 76 | 39.54 | 19.42 | 0.0820 |
| | Poor | 6 | 18.85 | 50.00 | |
| Dukes' stage | | | | | |
| | A | 7 | – | 0 | |
| | B | 52 | 40.57 | 17.31 | 0.0299 |
| | C | 28 | 25.33 | 32.14 | |
| sCD26 | | | | | |
| | ≤ 250 μg $l^{-1}$ | 52 | 33.73 | 26.92 | 0.0346 |
| | > 250 μg $l^{-1}$ | 35 | 38.97 | 11.43 | |

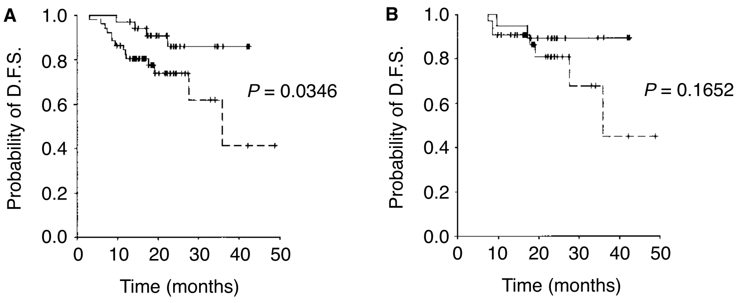

**Figure 4** Kaplan–Meier recurrence curves for colorectal cancer patients stratified by the preoperative serum CD26 levels in (**A**) total patients, and (**B**) patients of Dukes' B. Group1 (—) = Patients with sCD26 above 250 μg $l^{-1}$. Group 2 (---) = Patients with sCD26 equal or below 250 μg $l^{-1}$

stage were compared, sCD26 presents higher sensitivity than CEA to diagnose patients in Dukes' A, B and C stages. As recently reported, serum TIMP-1 (plasma tissue inhibitor of metalloproteinase) or sCD44v6 screened better only the Dukes' stage D CRC (Holten-Andersen et al, 1999; Yamane et al, 1999), and serum YKL-40 (Cintin et al, 1999) was clearly a poorer marker of diagnosis. Our conclusion is that preoperative sCD26 level is an useful, easy to handle marker for early detection of potentially curable CRC.

Reported recurrence rates after curative resection of large-bowel adenocarcinoma varied widely, partly because of how a recurrence is defined (Stipa et al, 1991), from 3% to 50%, usually within 2 years of surgery. As the risk factors commonly identified (level of invasion, lymphatic involvement, and site of original carcinoma) (Michelassi et al, 1990) do not always allow prediction of the outcome, which may guide the physician in aggressive but more selective adjuvant therapy and targeted surveillance in follow-up (Obrand and Gordon, 1997), we also studied if CD26 can help to distinguish CRC cases at high risk of tumour recurrence. Two groups of patients were differentiated by placing a cut-off point at the 60th percentile (log-rank test $P$ = 0.0346). According to our data there is no relationship between the preoperative serum sCD26 levels and the classical clinical features (Devesa et al, 1988). Meanwhile serum CEA levels, for example, correlate with the degree of histologic differentiation and the Dukes' stages classification. Thus new information about the prognosis of the patients is obtained. The different behaviour of sCD26 values in prognosis of Dukes' groups B and C can be also explained by the hypothesis explained above, because in C, the patients with an activation of the immune system (and thus with an

 *© 2000 Cancer Research Campaign*

enhancement in their sCD26 levels) might show a better survival than those with lower sCD26 values. The fact that analysis was performed in 2 years of recurrence, that the measure is not related with the pathological stage and can be carried out before the surgical operation, appears nevertheless to justify a follow-up of sCD26 as a prognostic variable.

### ACKNOWLEDGEMENTS

We thank Drs Mercedes Butrón and Gonzalo de Castro from the Hospital Xeral-Cíes de Vigo, and the Centro de Transfusión de Galicia for providing the blood samples. This work was supported by grants from Xunta de Galicia (XUGA 30110B97 and XUGA 20007B96) and from Universidade de Vigo. Daniel Ayude was supported by a predoctoral fellowship from 'Ministerio de Educación y Cultura', Spain.

### REFERENCES

American Society of Clinical Oncology (1996) Clinical practice guidelines for the use of tumor markers in breast and colorectal cancer. *J Clin Oncol* **14**: 2843–2877

American Society of Clinical Oncology (1998) 1997 update of recommendations for the use of tumor markers in breast and colorectal cancer. *J Clin Oncol* **16**: 793–795

Ansorge S, Schön E and Kunz D (1991) Membrane-bound peptidases of lymphocytes: functional implications. *Biomed Biochim Acta* **50**: 799–807

Ayude D, Rodríguez-Berrocal FJ, Fernándes-Rodrìguez J, Martínez-Zorzano VS, de Carlos A, Gil-Martin E and Páez de la Cadeno M (2000) Value of α-L-Fucosidase activity in the diagnosis of colorectal cancer. *Oncology* (in press)

Bauvois B (1988) A collagen-binding glycoprotein on the surface of mouse fibroblasts is identified as dipeptidyl peptidase IV. *Biochem J* **252**: 723–371

Bauvois B, Sancéau J and Wietzerbin J (1992) Human U937 cell surface peptidase activities: characterization and degradative effect on tumour necrosis factor-α. *Eur J Immunol* **22**: 923–930

Beck JR and Shultz EK (1986) The use of relative operating characteristic (ROC) curves in test performance evaluation. *Arch Pathol Lab Med* **110**: 13–20

Cintin C, Johansen JS, Christensen IJ, Price PA, Sorensen S and Nielsen HJ (1999) Serum YKL-40 and colorectal cancer. *Br J Cancer* **79**: 1494–1499

Cohn SL, Lincoln ST and Rosen ST (1986) Present status of serum tumour markers in diagnosis, prognosis and evaluation of therapy. *Cancer Invest* **4**: 305–327

Cordero OJ, Salgado FJ, Vinuela JE and Nogueira M (1997) Interleukin-12 enhances CD26 expression and dipeptidyl peptidase IV function on human activated lymphocytes. *Immunobiology* **197**: 522–533

Dang NH, Torimoto Y, Schlossman SF and Morimoto C (1990) Human VCD4 helper T cell activation: functional involvement of two distinct collagen receptors, 1F7 and VLA integrin family. *J Exp Med* **172**: 649–652

De Meester I, Korom S, Van Damme J and Scharpé S (1999) CD26, let it cut or cut it down. *Immunol Today* **20**: 367–375

De Meester I, Vanham G, Kestens L, Vanhoof G, Bosmans E, Gigase P and Scharpé S (1994) Binding of the adenosine deaminase to the lymphocyte surface via CD26. *Eur J Immunol* **24**: 566–570

Devesa JM, Morales V, Enriquez JM, Nuno J, Camunas J, Hernandez MJ and Avila C (1988) Colorectal cancer: the bases for a comprehensive follow-up. *Dis Colon Rectum* **31**: 636–652

Drucker DJ, Shi Q, Crivici A, Sumner-Smith M, Tavares W, Hill M, DeForest L, Cooper S and Brubaker PL (1997) Regulation of the biological activity of glucagon-like peptide 2 by dipeptidyl peptidase IV. *Nat Biotechnol* **15**: 673–677

Duke-Cohan JS, Morimoto C, Rocker JA and Schlossman SF (1996) Serum high molecular weight dipeptidyl peptidase IV (CD26) is similar to a novel antigen DPPT-L released from activated T cells. *J Immunol* **156**: 1714–1721

Dukes CE (1932) The classification of cancer of the rectum. *J Pathol Bacteriol* **35**: 323–332

Fernández-Rodríguez J, Ayude D, Páez de la Cadena M, Martínez-Zorzano VS, de Carlos A, Caride-Castro A, de Castro G and Rodríguez-Berrocal FJ (2000) α-L-Fucosidase enzyme in the prediction of colorectal cancer patients at high risk of tumor recurrence. *Cancer Detect* **24**: 143–149

Fleischer B (1994) CD26: a surface protease involved in T-cell activation. *Immunol Today* **15**: 180–184

Fujita K, Hirano M, Tokunaga K, Nagatsu I, Nagatsu T and Sakakibara S (1977) Serum glycylproline p-nitroanilidase activity in blood cancers. *Clin Chim Acta* **81**: 215–217

Gotoh H, Hagihara M, Nagatsu T, Iwata H and Miura T (1988) Activity of dipeptidyl peptidase IV and post-proline cleaving enzyme in sera from osteoporotic patients. *Clin Chem* **34**: 2499–2501

Gruss HJ, Pinto A, Duyster J, Poppema S and Herrmann F (1997) Hodgkin's disease: a tumour with disturbed immunological pathways. *Immunol Today* **18**: 156–163

Hakomori S (1989) Aberrant glycosylation in tumours and tumour-associated carbohydrate antigens. *Adv Cancer Res* **52**: 257–331

Hino M, Nagatsu T, Kakumu S, Okuyama S, Yoshii Y and Nagatsu I (1975) Glycylprolyl beta-naphthylamidase activity in human serum. *Clin Chim Acta* **62**: 5–11

Holten-Andersen MN, Murphy G, Nielsen HJ, Pedersen AN, Christensen IJ, Hoyer-Hansen G, Brunner N and Stephens RW (1999) Quantitation of TIMP-1 in plasma of healthy blood donors and patients with advanced cancer. *Br J Cancer* **80**: 495–503

Iwaki-Egawa S, Watanabe Y, Kikuya Y and Fujimoto Y (1998) Dipeptidyl peptidase IV from human serum: purification, characterization, and N-terminal amino acid sequence. *J Biochem (Tokyo)* **124**: 428–433

Iwata S and Morimoto C (1999) CD26/dipeptidyl peptidase IV in context. The different roles of a multifunctional ectoenzyme in malignant transformation. *J Exp Med* **190**: 301–306

Kameoka J, Tanaka T, Nojima Y, Schlossman SF and Morimoto C (1993) Direct association of adenosine deaminase with a T cell activation antigen CD26. *Science* **261**: 466–469

Kaplan EL and Meier P (1958) Nonparametric estimation from incomplete observations. *Am Stat Assoc* **53**: 457–481

Kojima K, Mihara R, Sakai T, Togari A, Matsui T, Shinpo K, Fujita K, Fukasawa K, Harada M and Nagatsu T (1987) Serum activities of dipeptidyl-aminopeptidase II and dipeptidyl-aminopeptidase IV in tumour-bearing animals and in cancer patients. *Biochem Med Metab Biol* **37**: 35–41

Lakatos PL, Firneisz G, Rakoczy G, Selmeci L and Szalay F (1999) Elevated serum dipeptidyl peptidase IV (CD26, EC 3.4.14.5) activity in patients with primary biliary cirrhosis. *J Hepatol* **30**: 740

Maes M, Lin A, Bonaccorso S, Vandoolaeghe E, Song C, Goossens F, De Meester I, Degroote J, Neels H, Scharpe S and Janca A (1999) Lower activity of serum peptidases in abstinent alcohol-dependent patients. *Alcohol* **17**: 1–6

Maes M, De Meester I, Verkerk R, De Medts P, Wauters A, Vanhoof G, Vandoolaeghe E, Neels H and Scharpe S (1997) Lower serum dipeptidyl peptidase IV activity in treatment resistant major depression: relationships with immune-inflammatory markers. *Psychoneuroendocrinology* **22**: 65–78

Medeiros MS and Turner AJ (1994) Post-secretory processing of regulatory peptides: the pancreatic polypeptide family as a model example. *Biochimie* **76**: 283–287

Michelassi F, Vannucci L, Ayala JJ, Chappel R, Goldberg R and Block GE (1990) Local recurrence after curative resection of colorectal adenocarcinoma. *Surgery* **108**: 787–792

Mogi M, Harada M, Hiraoka BY, Fukasawa K, Komatsu M and Nagatsu T (1986) Sandwich enzyme-immunoassay for dipeptidyl aminopeptidase IV in the serum of people with oral cancer. *Arch Oral Biol* **31**: 505–507

Morrison ME, Vijayasaradhi S, Engelstein D, Albino AP and Houghton AN (1993) A marker for neoplastic progression of human melanocytes is a cell surface ectopeptidase. *J Exp Med* **177**: 1135–1143

Nano R, Capelli E, Civallero M, Lorusso L, Argentina F, Bonizzoni E and Ceroni M (1997) Activated lymphoid cells in human gliomas: morphofunctional and cytochemical evidence. *Anticancer Res* **17**: 107–111

Obrand DI and Gordon PH (1997) Incidence and patterns of recurrence following curative resection for colorectal carcinoma. *Dis Colon Rectum* **40**: 15–24

O'Hara RJ, Greenman J, Drew PJ, McDonald AW, Duthie GS, Lee PW and Monson JR (1998) Impaired interleukin-12 production is associated with a defective anti-tumour response in colorectal cancer. *Dis Colon Rectum* **41**: 460–463

Oravecz T, Pall M, Roderiquez G, Gorrell MD, Ditto M, Nguyen NY, Boykins R, Unsworth E and Norcross MA (1997) Regulation of the receptor specificity and function of the chemokine RANTES (regulated on activation, normal T cell expressed and secreted) by dipeptidyl peptidase IV (CD26)-mediated cleavage. *J Exp Med* **186**: 1865–1872

Pangalos MN, Neefs JM, Somers M, Verhasselt P, Bekkers M, van der Helm L, Fraiponts E, Ashton D and Gordon RD (1999) Isolation and expression of novel human glutamate carboxypeptidases with N-acetylated alpha-linked acidic dipeptidase and dipeptidyl peptidase IV activity. *J Biol Chem* **274**: 8470–8483

Perner F, Gyuris T, Rakoczy G, Sarvary E, Gorog D, Szalay F, Kunos I, Szonyi L, Peterfy M and Takacs L (1999) Dipeptidyl peptidase activity of CD26 in serum and urine as a marker of cholestasis: experimental and clinical evidence. *J Lab Clin Med* **134**: 56–67

© 2000 Cancer Research Campaign 

Proost P, De Meester Y, Schols D, Struyf S, Lambeir A-M, Wuyts A, Opdenakker G, De Clercq E, Scharpé S and Van Damme J (1998) Amino-terminal truncation of chemokines by CD26/Dipeptidyl peptidase IV. Conversion of RANTES into a potent inhibitor of monocyte chemotaxis and HIV-1-infection. *J Biol Chem* **273**: 7222–7227

Schmitz T, Underwood R, Khiroya R, Bachovchin WW and Huber BT (1996) Potentiation of the immune response in HIV-1+ individuals. *J Clin Invest* **97**: 1545–1549

Sharpé S, De Meester Y, Vanhoof G, Hendriks D, Van Sand M, Van Camp K and Yaron A (1988) Assay of Dipeptidyl peptidase IV in serum by fluorimetry of 4-methoxy-2-naphthylamine. *Clin Chem* **34**: 2299–2305

Shibuya-Saruta H, Kasahara Y and Hashimoto Y (1996) Human serum dipeptidyl peptidase IV (DPPIV) and its unique properties. *J Clin Lab Anal* **10**: 435–440

Sliutz G, Tempfer C, Winkler S, Kohlberger P, Reinthaller A and Kainz C (1995) Immunohistochemical and serological evaluation of CD44 splice variants in human ovarian cancer. *Br J Cancer* **72**: 1494–1497

Stipa S, Nicolanti V, Botti C, Cosimelli M, Mannella E, Stipa F, Giannarelli D, Bangrazi C and Cavaliere R (1991) Local recurrence after curative resection for colorectal cancer: frequency, risk factors and treatment. *J Surg Oncol*: 155–160

Ten Kate J, Wijnen JT, Boldewijn J, Khan PM and Bosman FT (1985) Immunohistochemical localization of adenosine deaminase complexing protein in intestinal mucosa and in colorectal adenocarcinoma as a marker for tumour cell heterogeneity. *Histochem J* **17**: 23–31

Ten Kate J, Dinjens WN, Meera Khan P and Bosman FT (1986*a*) Adenosine deaminase complexing protein in cancer studies. *Anticancer Res* **6**: 983–988

Ten Kate J, van den Ingh HF, Khan PM and Bosman FT (1986*b*) Adenosine deaminase complexing protein (ADCP) immunoreactivity in colorectal adenocarcinoma. *Int J Cancer* **37**: 479–485

Uematsu T, Urade M, Yamaoka M and Yoshioka W (1996) Reduced expression of dipeptidyl peptidase (DPP) IV in peripheral blood T lymphocytes of oral cancer patients. *J Oral Pathol Med* **25**: 507–512

Uematsu T, Urade M and Yamaoka M (1998) Decreased expression and release of dipeptidyl peptidase IV (CD26) in cultured peripheral blood T lymphocytes of oral cancer patients. *J Oral Pathol Med* **27**: 106–110

Underwood R, Chiravuri M, Lee H, Schmitz T, Kabcenell AK, Yardley K and Huber BT (1999) Sequence, purification, and cloning of an intracellular serine protease, quiescent cell proline dipeptidase. *J Biol Chem* **274**: 34053–34058

Van Der Velden VH, Naber BA, Van Hal PT, Overbeek SE, Hoogsteden HC and Versnel MA (1999) Peptidase activities in serum and bronchoalveolar lavage fluid from allergic asthmatics – comparison with healthy non-smokers and smokers and effects of inhaled glucocorticoids. *Clin Exp Allergy* **29**: 813–823

Wesley UV, Albino AP, Tiwari S and Houghton AN (1999) A role for dipeptidyl peptidase IV in suppressing the malignant phenotype of melanocytic cells. *J Exp Med* **190**: 311–322

Werb Z (1997) ECM and cell surface proteolysis: regulating cellular ecology. *Cell* **91**: 439–442

Yamane N, Tsujitani S, Makino M, Maeta M and Kaibara N (1999) Soluble CD44 variant 6 as a prognostic indicator in patients with colorectal cancer. *Oncology* **56**: 232–238

Zweig MH and Campbell G (1993) Receiver-operating characteristic (ROC) plots: A fundamental evaluation tool in clinical medicine. *Clin Chem* **39**: 561–577

 *© 2000 Cancer Research Campaign*
